# Spatial timing of circulating seasonal influenza A and B viruses in China from 2014 to 2018

**DOI:** 10.1038/s41598-023-33726-7

**Published:** 2023-05-02

**Authors:** Ai-qin Zhu, Zhong-jie Li, Hang-jie Zhang

**Affiliations:** 1 Shanghai Mental Health Center, Shanghai Jiaotong University School of Medicine, Shanghai, 200025 People’s Republic of China; 2 Division of Infectious Disease, Key Laboratory of Surveillance and Early-Warning On Infectious Disease, Chinese Center for Disease Control and Prevention, Beijing, 102200 People’s Republic of China; 3 The Center for Disease Control and Prevention of Zhejiang Province, Hangzhou, Zhejiang 310051 People’s Republic of China; 4 Chinese Field Epidemiology Training Program, Chinese Center for Disease Control and Prevention, Beijing, 102200 People’s Republic of China

**Keywords:** Epidemiology, Population screening

## Abstract

Major outbreaks of influenza virus occurred in China in 2017–2018. To describe the pattern of influenza circulation and timing of seasonal epidemics, we analyzed data from influenza-like illness (ILI) specimens on surveillance wards of sentinel hospitals during 2014–2018. Among 1,890,084 ILI cases, 324,211 (17.2%) tested positive for influenza. Influenza A virus (particularly A/H3N2), which circulates annually, was detected in 62% of cases, compared with influenza B virus in 38% of cases. The detection rate of A/H1N1, A/H3N2, B/Victoria, and B/Yamagata viruses were 3.56%, 7.07%, 2.08%, and 3.45%, respectively. Influenza prevalence was generally stable over the four years analyzed, but obvious outbreaks occurred in 2015–2016 (17.28%) and 2017–2018 (22.67%), with B/Victoria and B/Yamagata contributing to these outbreaks, respectively. In the south, a characteristic peak in infections was detected in the summer (week 23–38), which was not detected in the north. Influenza B was found high frequency in school-age children (5–14 years) with 4.78% of B/Victoria and 6.76% of B/Yamagata. Therefore, the epidemiological characteristics of seasonal influenza were complex in China during 2014–2018, presenting distinctions in region, season, and susceptible population. These findings underline the importance of enhancing year-round influenza surveillance and provide a reference for the timing and variety of influenza vaccination.

## Introduction

Seasonal influenza is an acute respiratory illness caused by the influenza virus. It is responsible for annual, seasonal epidemics worldwide that cause a substantial burden on healthcare services, despite decades of surveillance and pharmaceutical and non-pharmaceutical interventions^1^. The World Health Organization (WHO) estimates that the influenza virus causes 3 to 5 million severe infections and 290,000 to 650,000 fatal infections annually worldwide^2^. In China, the number of annual influenza-associated excess deaths was 18.0 and 11.3 per 100,000 in northern and southern cities, respectively^3^. Viruses of influenza A and B lineages are the main causes of the disease burden in humans^4^. The epidemic strength of influenza B is lower than that of influenza A in China. However, influenza B is more prevalent in some regions in certain years, and the B/Yamagata and B/Victoria lineages were reported to be alternately dominant in the winter-spring months^5^.

Throughout the world, influenza shows seasonal prevalence and high incidence in temperate regions in the winter and spring. However, in tropical regions, especially in Asia, influenza seasonality is highly diverse, with semi-annual and year-round epidemics, as well as year-round cycles^6–8^. China is a country with a vast territory encompassing a diversity of climatic zones (temperate, subtropical, and tropical), and factors such as the minimum temperature, hours of sunshine, and maximum rainfall affect influenza seasonality. The annual periodicity of influenza A increased with latitude and showed diversified spatial patterns and seasonal characteristics. Epidemics of influenza A peaked in January–February in northern China (latitude ≥ 33°N) and April–June in southernmost regions (latitude < 27°N), and dominant semi-annual periodicity was observed with peaks in January–February and June–August (latitude 27.4°N–31.3°N)^9^. Despite the vast amount of study on seasonal influenza, the epidemiological characteristics of seasonal influenza have not been well described on a national scale in China. Consequently, analyzing surveillance data with different populations, periods, and regions of specific influenza types, subtypes, and lineages is crucial to develop policies and strategies for the prevention and control of the influenza epidemic, as well as understanding influenza persistence worldwide.

China established an influenza surveillance system in 2000, which is conducted in 31 provinces (autonomous regions or municipalities) in mainland of China, under the management of the China Center for Disease Control and Prevention (China CDC), providing weekly reports from 410 laboratories and 554 sentinel hospitals in each province^10^. In this study, we used the results of weekly viral surveillance of influenza surveillance wards of sentinel hospitals to estimate the influenza case rate in China between 2014 and 2018. We identified and compared the seasonality and epidemiological features of seasonal influenza subtypes, as well as regional differences between north and south China. Based on our findings, we provide guidance on developing specific vaccine recommendations and on the prevention and control of influenza.

## Results

### Overall characteristics of influenza-positive cases

﻿From 2014–2018, 1,890,084 outpatients or inpatients diagnosed with influenza-like illness (ILI) cases were enrolled ﻿for specimen collection in the surveillance wards of the sentinel hospitals. Of these, 324,211 ILI patients were diagnosed with laboratory-confirmed influenza, and 173,119 (53.4%) were male. The median age was 12.0 years (interquartile range [IQR], 5.0–33.0 years), 86,713 (26.7%) were 0–4 years old, 107,774 (33.2%) were ﻿5–14 years old, 34,165 (10.5%) were 15–24 years old, 74,323 (22.9%) were 25–59 years old, and 21,236 (6.6%) were ≥ 60 years old (Table 1). The median age of ILI patients with confirmed influenza was 12.0 years, compared to 8.0 years in those without confirmed influenza.

**Table 1:** The frequency of influenza viruses and proportion for influenza type /subtype by subgroups.

Characteristics	No. samples tested	No.(%) of influenza cases	No.(proportion) of influenza cases						
			A (Not subtyped)	A/H1N1	A/H3N2	B (Not subtyped)	B/Victoria	B/Yamagata	Coinfection
Year									
2014–2015	420,067	55,610 (13.24)	86 (0.02)	917 (0.22)	37,650 (8.96)	4125 (0.98)	414 (0.10)	12,320 (2.93)	98 (0.02)
2015–2016	461,136	79,689 (17.28)	55 (0.01)	16,152 (3.5)	26,756 (5.8)	9741 (2.11)	17,916 (3.89)	8951 (1.94)	118 (0.03)
2016–2017	450,698	62,396 (13.84)	44 (0.01)	10,871 (2.41)	38,204 (8.48)	2128 (0.47)	9593 (2.13)	1529 (0.34)	27 (0.01)
2017–2018	558,183	126,516 (22.67)	24 (0.00)	39,428 (7.06)	30,971 (5.55)	1986 (0.36)	11,385 (2.04)	42,384 (7.59)	338 (0.06)
Sex									
Male	1,019,634	173,119 (16.98)	120 (0.01)	35,924 (3.52)	70,926 (6.96)	10,067 (0.99)	21,104 (2.07)	34,643 (3.4)	335 (0.03)
Female	870,450	151,092 (17.36)	89 (0.01)	31,444 (3.61)	62,655 (7.2)	7913 (0.91)	18,204 (2.09)	30,541 (3.51)	246 (0.03)
Age group (years)									
0–4	744,125	86,713 (11.65)	74 (0.01)	21,604 (2.9)	37,298 (5.01)	4943 (0.66)	9893 (1.33)	12,751 (1.71)	150 (0.02)
5–14	416,699	107,774 (25.86)	47 (0.01)	18,633 (4.47)	32,640 (7.83)	8106 (1.95)	19,910 (4.78)	28,185 (6.76)	253 (0.06)
15–24	173,993	34,165 (19.64)	23 (0.01)	5599 (3.22)	17,257 (9.92)	1342 (0.77)	3056 (1.76)	6842 (3.93)	46 (0.03)
25–59	433,081	74,323 (17.16)	52 (0.01)	17,613 (4.07)	35,168 (8.12)	2827 (0.65)	5687 (1.31)	12,881 (2.97)	95 (0.02)
≥ 60	122,186	21,236 (17.38)	13 (0.01)	3919 (3.21)	11,218 (9.18)	762 (0.62)	762 (0.62)	4525 (3.7)	37 (0.03)
Region									
Northern hina	694,317	115,031 (16.57)	9 (0)	24,935 (3.59)	47,990 (6.91)	7904 (1.14)	11,097 (1.6)	22,829 (3.29)	267 (0.04)
Southern China	1,195,767	209,180 (17.49)	200 (0.02)	42,433 (3.55)	85,591 (7.16)	10,076 (0.84)	28,211 (2.36)	42,355 (3.54)	314 (0.03)
Specimens									
URT	1,889,431	324,067 (17.15)	206 (0.01)	67,275 (3.56)	133,554 (7.07)	17,978 (0.95)	39,306 (2.08)	65,170 (3.45)	578 (0.03)
LRT	653	144 (22.05)	3 (0.46)	93 (14.24)	27 (4.13)	2 (0.31)	2 (0.31)	14 (2.14)	3 (0.46)
Total	1,890,084	324,211 (17.15)	209 (0.01)	67,368 (3.56)	133,581 (7.07)	17,980 (0.95)	39,308 (2.08)	65,184 (3.45)	581 (0.03)

*URT* Upper respiratory tract specimens; *LRT* Lower respiratory tract specimens.

Across the four seasons, the confirmed average annual number of influenza infections was 81,053 (95% CI: 30,199, 131,907), corresponding to a detected positive rate of 17.2% (95% CI: 9.9% to 23.6%) in ILI cases (Table 1). Influenza types/subtypes detected included A/H1N1(pH1N1) influenza (67,368, 3.56%), A/H3N2 (133,581, 7.07%), A (subtype not identified) (209, 0.01%), B/Victoria (39,308, 2.08%), B/Yamagata (65,184, 3.45%), B (subtype not identified) (17,980, 0.95%), and co-infection (581, 0.03%). Additionally, influenza-positive samples were detected with a percentage of 22.05% in lower respiratory tract specimens and 17.15% in upper respiratory tract specimens.

### Seasonal characteristics of influenza viruses

**Figure 1:**
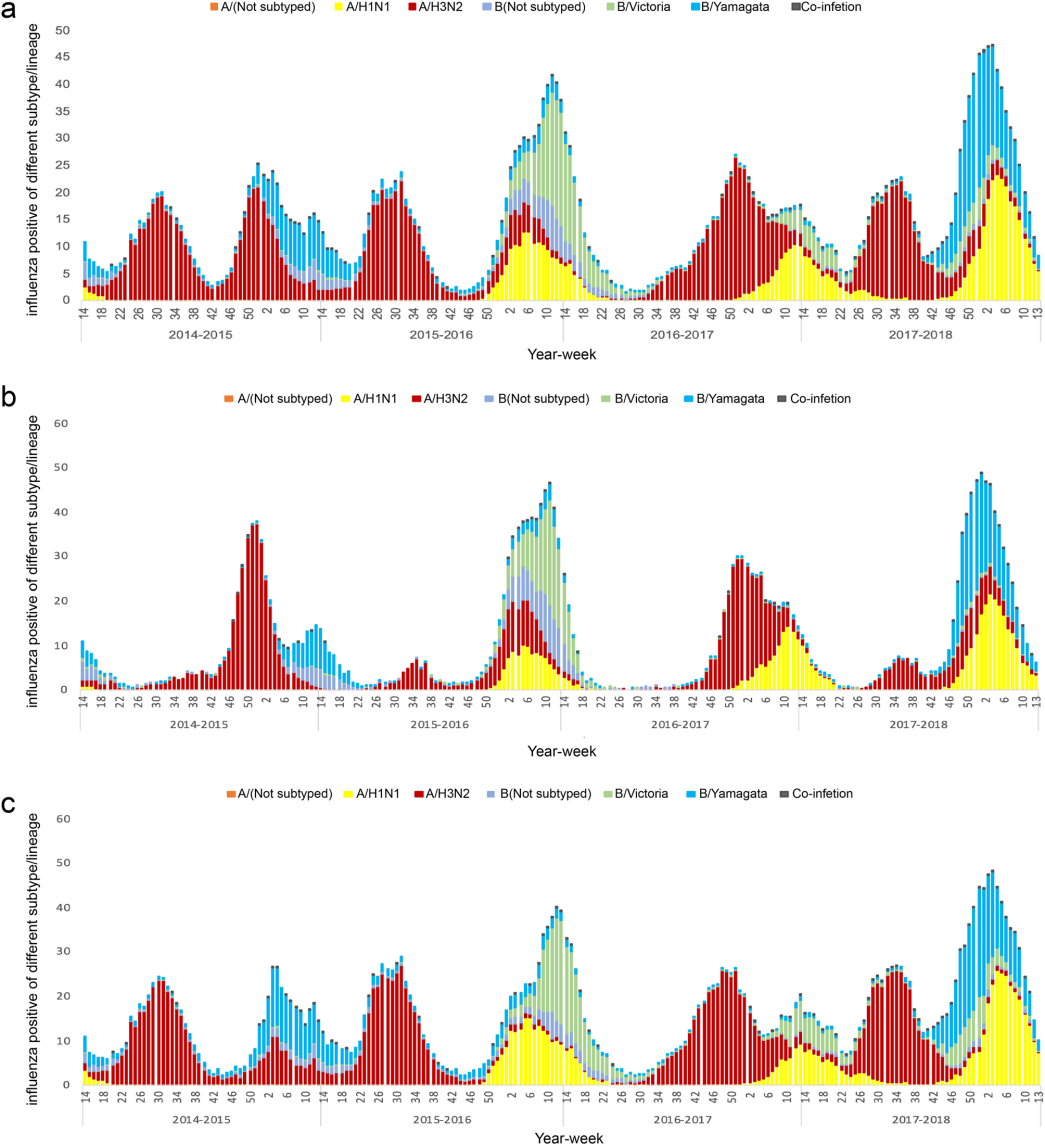
Weekly distribution of the positive detection rate of influenza virus from 2014 to 2018. (**a**: National distribution; **b**: Distribution in northern China; **c**: Distribution in southern China).

During the four-year surveillance period, we found three epidemic periodicities of the seasonal influenza virus. The first pattern was the annual periodicity of infection cases during winter. In the surveillance year 2016–2017, the estimated peak of influenza-positive detection rate of the weekly count was 25.1% in week 50. The second pattern was the semiannual periodicity of infection cases during the surveillance years 2014–2015 and 2015–2016, which presented two peaks of influenza-positive detection rate in the summer and in the winter-spring. For these two time periods, the influenza epidemic in the summer season peaked at week 30 with a positive detection rate of 20.1% and 22.3%, while the epidemic in the winter-spring peaked at weeks 52 and 12 with a positive detection rate of 25.1% and 41.8% for the years 2014–2015 and 2015–2016, respectively. The third pattern was the year-round periodicity of infection cases during the surveillance year 2017–2018. The influenza epidemic continued in the spring of the previous year, then peaked twice in the summer and winter-spring. The estimated summer peak was in week 33 and the winter peak was in week 4, and the positive detection rate at these peak times was 21.2% and 48.3%, respectively (Fig. 1a).

The positive detection rate of the influenza virus was undulatory according to seasonality. The highest influenza-positive detection rate occurred in winter (weeks 50–11), followed by summer (weeks 23–38), spring (weeks 12–22), and autumn (weeks 39–48). The epidemic of influenza A occurred in summer and winter, while the epidemic of influenza B occurred in winter and spring. With semiannual periodicity, influenza virus A/H3N2 was the most prevalent in the summer, peaking at a weekly positive rate of 20.1%–22.3%. Additionally, in winter-spring, A/H1N1 or influenza B were the most prevalent viruses after the peak of A/H3N2 at the same periodicity. A/H1N1 was mainly detected in winter-spring accompanied by influenza B viruses, such as B/Victoria in 2015–2016 and B/Yamagata in 2017–2018. B lineage identification occurred without regularity, with epidemic outbreaks of a particular lineage being detected every few years, e.g., 2015–2016 and 2017–2018 (Fig. 1a).

### Characteristics of influenza epidemics by region

**Figure 2:**
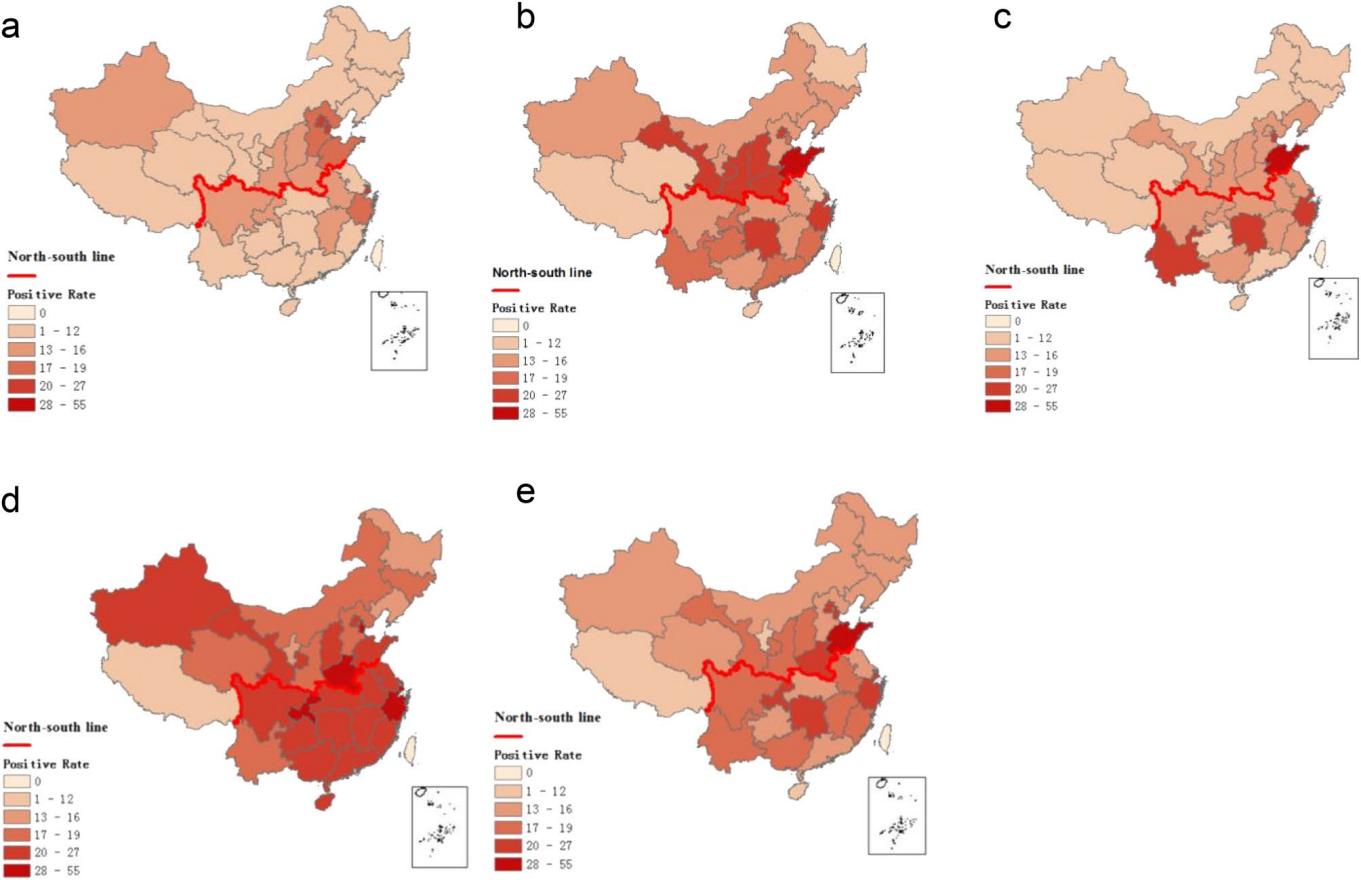
Distribution of the positive detection rate of influenza virus among provinces for different years. (**a**: 2014–2015; **b**: 2015–2016; **c**: 2016–2017; **d**: 2017–2018; **e**: 2014–2018). The maps were generated using ArcGIS (version 10.2, URL https://www.esri.com/).

The characteristics of influenza epidemics vary between the north and south of China, which is likely due to the wide range of climatic zones spanning from the northern mid-temperate region to the southern subtropical and tropical regions^5,11^. Local influenza outbreaks were mainly recorded in the northern provinces, e.g., Shandong, Henan, Shanxi, and Gansu in 2015–2016; however, in 2016–2017, influenza was prevalent in both northern and southern provinces (Fig. 2). During the outbreaks from 2014–2018, the most affected provinces in mainland China were Beijing, Tianjin, Shandong, Henan, Hunan, and Zhejiang.

**Figure 3:**
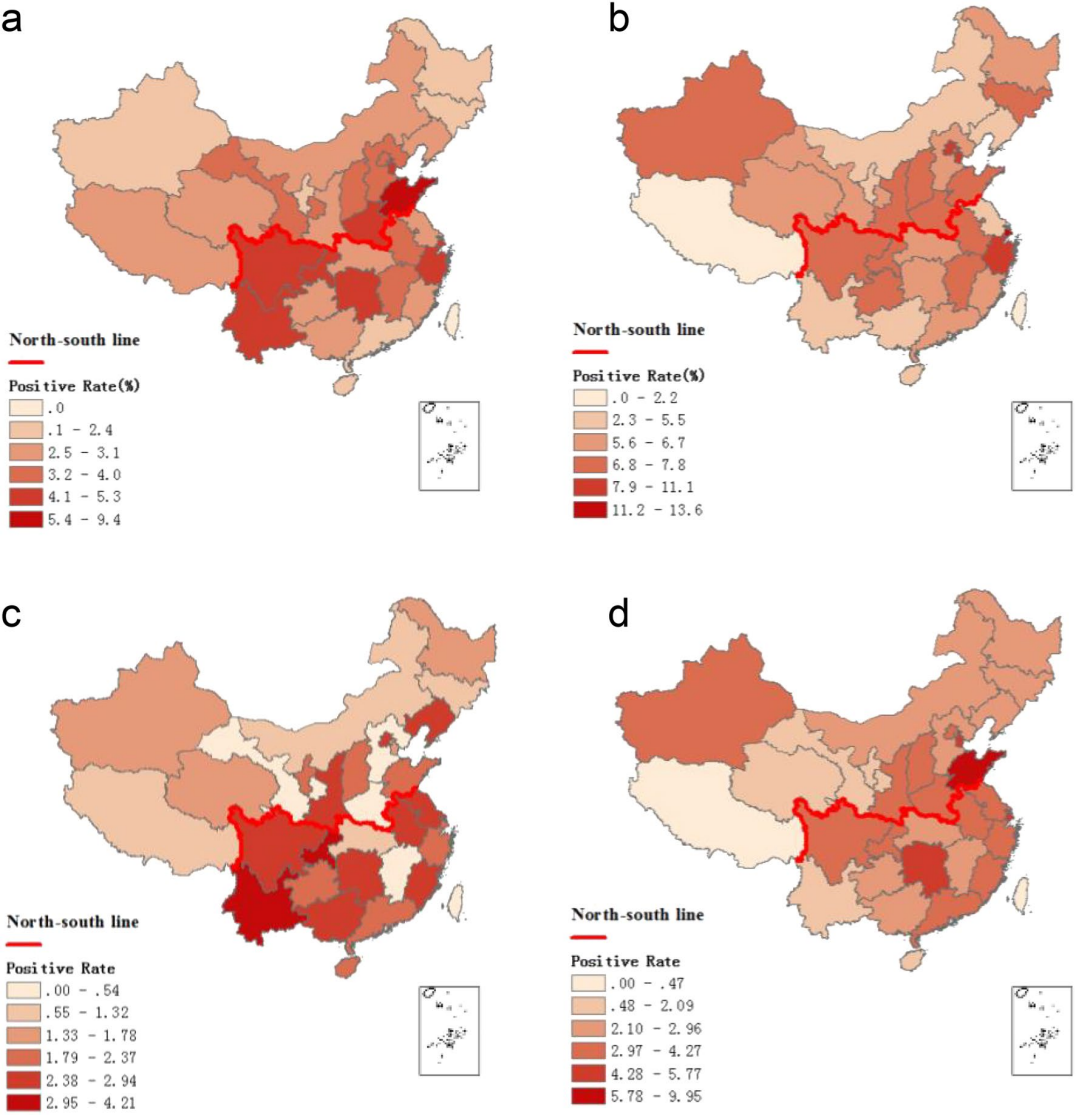
Distribution of the positive detection rates of different subtypes/lineages among provinces. (**a**: A/H1N1; **b**: A/H3N2; **c**: B/Victoria; **d**: B/Yamagata). The maps were generated using ArcGIS (version 10.2, URL https://www.esri.com/).

In northern China, there was an increase in influenza activity from December to May, presenting as a winter-spring seasonal peak during 2014–2018. Additionally, the predominant types/subtypes of influenza virus in the north were different for each seasonal peak of influenza activity (Fig. 1b). Virus A/H3N2 was predominant during the winter peak (accounting for 52% of positive samples) and B/Yamagata was predominant during the spring peak (accounting for 27% of positive samples) during 2014–2015. Viruses A/H1N1 and A/H3N2 were detected simultaneously with B/Victoria or B/Yamagata (accounting for 43% or 25% of positive samples) during the 2015–2016 and 2017–2018 winter peaks, respectively. Interestingly, only A/H1N1 (accounting for 49% of positive samples) and A/H3N2 (accounting for 40% of positive samples) predominated during the winter peak or the winter-spring peak in 2016–2017, with no detected outbreak of influenza B virus (Figs. 1b and 3).

In southern China, influenza activity among ILI patients peaked in both the winter-spring months and the summer months (weeks 23 to 38) each year. The predominant types/subtypes of circulating influenza virus in the south for the summer months of each influenza season were the same, mainly involving A/H3N2. Similar to the north, the predominant types/subtypes of circulating influenza virus in the south for both the winter and spring months of each influenza season varied from season to season (Fig. 1c and Fig. 3). During the winter-spring months of the study period, the types/subtypes of influenza virus were A(H3N2) and B/Yamagata during 2014–2015 (accounting for 34% and 60% of positive samples, respectively); A(H1N1) and B/Victoria during 2014–2015 (accounting for 56% and 40% of positive samples, respectively) and 2016–2017 (accounting for 47% and 38% of positive samples, respectively); and A(H1N1), B/Yamagata, and B/Victoria during 2017–2018 (accounting for 61%, 28%, and 10% of positive samples, respectively).

### Influenza virus activity among age groups

**Figure 4:**
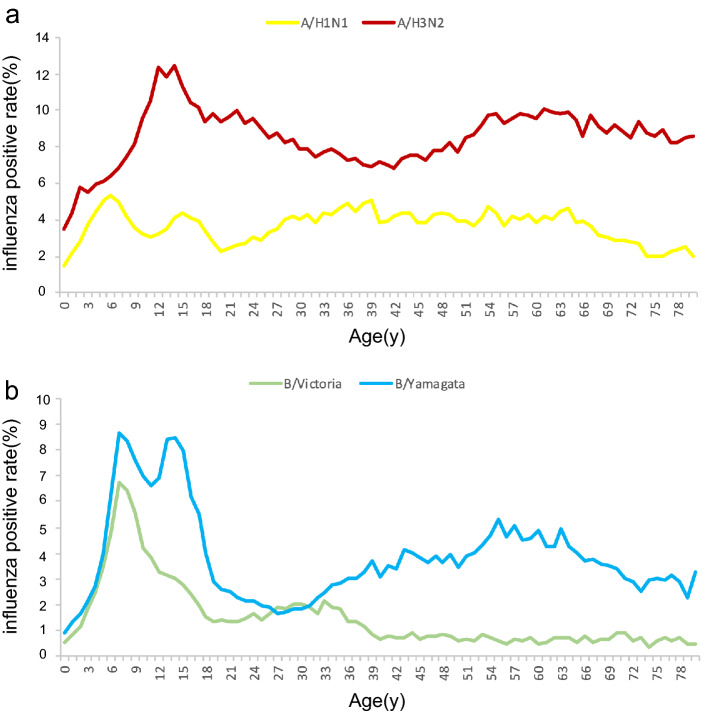
Correlation between age and the proportion of specimens testing positive for influenza, from 2014–2018. (**a**) A/H1N1 and A/H3N2; (**b**) B/Victoria and B/Yamagata lineages.

During the four-year period analyzed, the positive detection rate of influenza virus varied by age group, with the highest overall rate being among school-age children of 5–14 years old (25.86%), followed by 15–24-year-olds (19.64%), ≥ 60-year-olds (17.38%), 25–59-year-olds (17.16%), and 0–4-year-old (11.65%). For the influenza A virus, the positive detection rate of A/H3N2 (7.07%) and A/H1N1 (3.56%) among all age groups. The age distribution curve showed that the A/H1N1 positive detection rate remained relatively constant, whereas A/H3N2 showed two obvious peaks in 15–24-year-olds and ≥ 60-year-olds, at almost one-third of the rate of A/H1N1 (Fig. 4a). For influenza B viruses, the positive detection rate of B/Victoria and B/Yamagata peaked in the 5–14-year-old group (4.78% and 6.76%, respectively). The age distribution curve showed a single peak for B/Victoria, and this virus was rarely detected among those aged ≥ 40. However, detection of B/Yamagata decreased in adults ≥ 18 years of age, then increased again in the 40-year-old age group, and finally peaked among 54–63-year-olds (Fig. 4b).

## Discussion

In this study, we compared the relative contribution of the different virus types and subtypes to influenza epidemics and investigated the spatial–temporal patterns of seasonal influenza activity in northern and southern China during four consecutive seasons (2014–2015 to 2017–2018). On average, A/H3N2 caused nearly one-half of all cases and was epidemic in all four seasons. The circulation patterns of the other three main influenza viruses differed substantially from those of A/H3N2, and their contributions to seasonal epidemics and dynamic patterns need to be further studied. Influenza B virus caused major outbreaks in two of the four seasons (2015–2016 and 2017–2018), confirming its role as an important contributor to the seasonal influenza burden, which has similarly been observed in other regions of the world^12^. In particular, B/Victoria and B/Yamagata made a significant contribution to the influenza burden in 2015–2016 and 2017–2018, respectively. Understanding the epidemiological characteristics of the two influenza B virus lineages will provide valuable insights into the appropriate modification of current influenza vaccination programs.

We identified three epidemic periodicities of seasonal influenza viruses during the surveillance period from 2014–2018, including annual periodicity, semiannual periodicity, and year-round periodicity. The epidemic durations for viruses with annual periodicity were longer than those with semiannual periodicity; epidemics with annual periodicity lasted for 38 weeks (week 34 to 12) in the winter-spring seasons, while epidemics with semiannual periodicity lasted for 16 weeks (week 22 to 38) in the summer seasons and 20 weeks (week 50 to 18) in the winter-spring seasons. In addition, the epidemic with year-round periodicity continued from week 14 of the previous season in winter and spring, and epidemics with semiannual periodicity continued for another influenza season. This finding highlights the importance of understanding influenza circulation patterns in China.

Seasonal influenza infections exhibit a strong seasonal cycle, both according to the longitude and latitude, in most cases. Distinct seasonality has previously been reported in China: northern provinces (latitudes > 33°N) experience winter epidemics, southernmost provinces (latitude < 27°N) experience peak activity in spring, while provinces at intermediate latitudes experience semi-annual epidemic cycles^13^. On the basis of the influenza surveillance data from 2014–2018, we identified similar epidemiological characteristics, with southern China having earlier primary peaks in the winter-spring months and an obvious secondary peak in the summer months, whereas northern China had one peak of similar amplitude in the winter-spring months each year. Despite the observed differences in latitude, there remained uncertainty regarding the main driver of the influenza epidemics. Climatic parameters, such as absolute humidity, temperature, and rainfall, cause differences in influenza epidemiology (including the timing, periodicity, and patterns of transmission). Recent laboratory and epidemiological evidence suggests that low humidity conditions facilitate the airborne survival and transmission of the influenza virus in temperate regions, resulting in annual winter epidemics^14^.

Published data from 29 countries from 1999 to 2014 suggested that young children (< 5 years of age) were more affected by seasonal influenza A/H1N1, older children (aged 5 to 17 years) by influenza B, young adults (aged 18 to 39 years) and older adults (aged 40 to 64 years) by influenza A/H1N1, and older people (aged ≥ 65 years) by influenza A/H3N2^15^. Our study indicated that influenza positivity was high among school-age children (5 to 14-year-olds), especially for B/Victorian and B/Yamagata. This revealed that influenza B virus infections are of specific concern among pediatric patients because of the increased risk of severe disease^16^. Future research should prioritize the study of influenza epidemiology in this age-specific population, to optimize prevention strategies and the composition and timing of administration of the influenza vaccine. Additionally, the current trivalent influenza vaccine (TIV) is lack of B/Y antigen and quadrivalent influenza vaccines (QIV) should be timely updated for better prevention against type B influenza virus.

Despite the availability of influenza surveillance data greatly improving since the 2009 pandemic, there are still some gaps in the data that may be seen as limitations of the present study. First, the number of reported influenza cases (in each season and overall) varied widely across provinces, and this influenced our analysis of the regional characteristics of influenza epidemics. Second, we did not analyze the effects of climatic parameters (humidity, temperature, and rainfall) on influenza epidemiology. Third, the detailed co-infection distribution of different influenza viruses is not available in our database. Moreover, due to the update of influenza surveillance guidelines in 2017, and the improvement of detection technology capability from 2014 to 2018, may affect the positive rate of influenza detection.

In conclusion, our study uncovered the intriguing characteristics of influenza seasonality and defined the predominant circulating virus types and subtypes in different regions of China. Furthermore, our findings revealed patterns of age‐specific susceptibility to influenza infection. Such information is valuable for estimating the disease burden of influenza and evaluating the target groups and optimizing the timing of vaccination and other preventive measures against influenza. Further surveillance studies are warranted to confirm these seasonality patterns and to ensure that the influenza strains circulating in different provinces are consistent with WHO vaccine recommendations.

Methods.

### Epidemiological data

Data of outpatients or inpatients diagnosed with ILI were collected from the national influenza surveillance network from 2014–2018 and included sex, age, date of sampling, respiratory specimen type, the number of laboratory-confirmed influenza cases by virus type (influenza A and B), and the number of ILI specimens tested in 31 Chinese provinces. ILI was defined as a measured temperature ≥ 38 °C with the presence of either cough or sore throat. Hospital infection control and local CDC staff entered epidemiologic, clinical, and laboratory data into an electronic database that was transmitted weekly to the national China CDC ^3,11^. All methods were carried out in accordance with relevant guidelines and regulations. Participating hospitals and laboratories used a standard operating protocol (SOP) of surveillance that included guidelines for patient enrollment, specimen collection, laboratory testing, data recording, and management, developed by China CDC^17^.

National Health Commission of the People’s Republic of China decided that since data from patients with ILI was part of continuing public health surveillance and implemented national surveillance guidelines. All participants and/or their legal guardian(s) in this study agreed with an informed consent to provide information such as sex, age and area, and specimen testing for influenza during their enrollment. This project and the above procedure for obtaining consent were approved by the ethical review committee of the Chinese Center for Disease Control and Prevention (China CDC, Beijing, China).

### Specimen collection and testing

After hospital admission and verbal consent from eligible ILI cases- or their parent/guardians, ﻿physicians collected the upper respiratory tract specimens (nasopharyngeal swab or pharyngeal swab) and lower respiratory tract specimens (aspirate, sputum, bronchoalveolar lavage, or lung puncture aspirate) within 24 h of enrollment, following standardized procedures. Specimens were immediately placed in a viral transport medium (VTM) and stored at 4 °C at the local hospital. These specimens were transferred to the closest influenza network laboratory (provincial or prefecture CDC laboratories) within 48 h of collection. The local influenza network laboratories stored the specimens in VTM at − 70 °C prior to reverse transcription PCR (RT-PCR), or virus isolation and detection using a hemagglutination-inhibition test (HI test), following the standard protocol^17^. We defined a patient with laboratory-confirmed influenza as any ILI patient with a ﻿respiratory specimen that tested positive for influenza virus by RT-PCR or HI test. If any one of the influenza viruses was detected in the specimens, the patient was considered to be positive. One virus identified was labeled as a single infection, and two or more etiologies were co-infection.

### Data analysis

The positive detection rate was calculated by dividing the number of samples that tested positive for the influenza virus by the total number of samples tested. The age-specific influenza positive detection rate by subtype/lineage was calculated as the number of specimens that tested positive for each subtype/lineage (numerator) among ILI cases recruited for specimen collection (denominator) in each corresponding age group^18^. A surveillance year was defined as the period ranging from calendar week 14 of one year to calendar week 13 of the next year^17^. The surveillance week started on Monday and ended on Sunday. The 12th week to the 22nd week of each year is spring, the 23rd week to the 38th week is summer, the 39th week to the 48th week is autumn, and the 50th week to the 11th week of the following year is winter. The start of an influenza epidemic period was defined as the first week during which the positive rate of influenza virus testing was higher than 10% and stayed above that level for at least four consecutive weeks. The end of an influenza epidemic period was defined as the first week during which the positive rate was lower than 10% and stayed at that level for at least four consecutive weeks^19^. Based on influenza activity, climate, and geography, we divided China into two distinct regions, north and south, for the analysis and interpretation of influenza surveillance data. The Qinling Mountain–Huaihe River serves as the dividing line between north and south China^20^. Data were performed using Microsoft Excel (Version 2021, Microsoft Corporation, Redmond, WA, USA), Stata version 11 (StataCorp LP, College Station, TX, USA) and ArcGIS (version 10.2, ESRI Inc.; Redlands, CA, USA; URL https://www.esri.com/).

## Data Availability

The datasets generated and analyzed during this study are not publicly available owing to the institute’s data security and sharing policy, but they are available from the corresponding author upon reasonable request.
